# HuD and alpha-crystallin A axis protects neuro-retinal cells in early diabetes

**DOI:** 10.1007/s11010-025-05364-2

**Published:** 2025-08-12

**Authors:** Chongtae Kim, Subeen Oh, Young-Hoon Park

**Affiliations:** 1 Catholic Institute for Visual Science, College of Medicine, The Catholic University of Korea, Seoul, 06591 South Korea; 2 Department of Ophthalmology, College of Medicine, The Catholic University of Korea, Seoul, 06591 South Korea; 3 Department of Ophthalmology and Visual Science, Seoul St. Mary’s Hospital, 222 Banpodaero, Seocho-Gu, Seoul, 06591 South Korea

**Keywords:** HuD, CRYAA, Retina, Diabetic retinopathy, Ganglion cells

## Abstract

Diabetic retinopathy (DR) is a prevalent microvascular complication of diabetes; however, neuro-retinal degeneration is also observed in patients with diabetes without signs of DR. The mechanisms leading to neuro-retinal cell loss before vascular complications manifest in diabetes remain poorly understood. In this study, we investigated the neuronal RNA-binding protein HuD as a novel regulator of neuro-retinal degeneration in the early stage of diabetes. We determined the expression of HuD and alpha-crystallin A (CRYAA) in the retinal ganglion cell layer. HuD and CRYAA were down-regulated in the retinas of streptozotocin-induced diabetic rats and in neuro-retinal cells (R-28) treated with high glucose. *Cryaa* mRNA was identified as a novel target transcript of HuD, and we demonstrated that HuD post-transcriptionally regulates the expression of *Cryaa* mRNA by binding to its 3′-untranslated region. Silencing and overexpression of HuD positively regulated the expressions of *Cryaa* mRNA and protein. We demonstrated that the increase in inflammatory cytokines such as TNFα, IL-1β, and IL-6 in R-28 cells under hyperglycemic conditions was a result of both CRYAA and HuD levels. Silencing HuD and CRYAA enhanced high glucose-induced R-28 cell death, whereas their overexpression alleviated this effect. HuD post-transcriptionally regulates CRYAA expression, influencing the function and viability of neuro-retinal cells under diabetic conditions. Our results suggest that the HuD/CRYAA axis plays a crucial role in neuro-retinal cells and has the potential to serve as a prognostic factor and therapeutic target for diabetic neuro-retinal degeneration.

## Introduction

Diabetic retinopathy (DR) is a common microvascular complication of diabetes mellitus and a significant cause of vision loss, particularly in middle-aged and older populations. The progression of DR is classified into various levels of severity, including mild, moderate, and severe non-proliferative DR (NPDR) and proliferative DR (PDR), based on the extent of vascular lesions [1]. Vascular abnormalities are the most apparent signs for diagnosing and treating DR; however, these phenotypes manifest in the later stages of the disease. An increasing number of studies have emphasized that neuronal cells in the retina are affected during early diabetes, leading to the degeneration of neuro-retinal cells, such as retinal ganglion cells (RGCs) [2]. Even patients with diabetes without DR exhibit neuro-retinal alterations, including ganglion cell-inner plexiform layer (GC-IPL) loss [3, 4], thinning of the macular ganglion cell complex thickness [5, 6], and changes in RGCs [7]. Although neuro-retinal degeneration is observed in patients with early diabetes without any signs of DR, the precise molecular mechanism yet remains to be elucidated.

HuD is an RNA-binding protein that regulates mRNA stability and translation by binding to adenylate/uridylate-rich elements (AREs) in target mRNAs [8]. In many cases, the role of HuD in neuronal function, maintenance, and development has been revealed because its predominant expression in neuronal cells [9, 10]. In addition to its reported role in insulin synthesis [11], HuD has been shown to play a key role in pancreatic β cells, where it is involved in autophagosome formation, lipid metabolism, cell cycle progression, and mitochondrial dynamics [12]. Although the retina is composed of diverse neuronal cells in ocular tissue, the role of HuD has not yet been elucidated in detail and remains poorly studied.

Crystallins are water-soluble structural proteins found in lenses. Since their discovery in the lens, crystallins have been identified in various tissues, including the retina, heart, skeletal muscles, skin, brain, and others [11]. Crystallins are classified into three main types: alpha-, beta-, and gamma-crystallins. Alpha-crystallins possess chaperone-like properties, preventing the precipitation of denatured proteins and enhancing cellular tolerance to diverse cellular stresses [12].

Several studies have clarified the implications of crystallin expression in DR. The levels of alpha-crystallin A (CRYAA) are significantly down-regulated in the retinal tissues of streptozotocin (STZ)-induced diabetic mice [13] and STZ-induced rats [14]. Fort et al. demonstrated that the protein synthesis of *Cryaa* mRNA is down-regulated in the retinas of diabetic rats using polysome fractionation assays [15]. However, the molecular mechanism underlying CRYAA expression in diabetic retinal tissue has not yet been fully elucidated.

Here, we identified the roles of HuD expressed in retinal ganglion cells. Both HuD and CRYAA were downregulated in diabetic retinal tissues and neuro-retinal cells. *Cryaa* mRNA was identified as a novel downstream target of HuD. Our results elucidate the molecular mechanism linking HuD and CRYAA under diabetic conditions and the potential role in ameliorating the degeneration of neuro-retinal cells.

## Materials and methods

### Cell culture, treatment, and transfection of siRNAs and plasmids

The immortalized rat retinal precursor cell line, R-28, was cultured in Dulbecco’s Modified Eagle’s Medium (DMEM; Welgene, Gyeongsan-si, South Korea) supplemented with 10% fetal bovine serum (FBS; HyClone, UT, USA), 1% penicillin (Welgene), and normal glucose (5.5 mM). Cells were maintained at 37 °C in a humidified incubator with 5% CO₂. For experimental treatments, the cells were exposed to a high glucose condition (25 mM glucose; Sigma-Aldrich, St. Louis, MO, USA). Plasmid transfection was performed using Lipofectamine™ 2000 (Invitrogen, Carlsbad, CA, USA). The constructs included an empty vector (pcDNA), myc-tagged HuD (pHuD), myc-tagged CRYAA (pCRYAA), and enhanced green fluorescent protein reporter (pEGFP). For gene silencing studies, small interfering RNAs (siRNAs) comprising control siRNA (siCtrl), HuD-targeted siRNA (siHuD), and Cryaa-targeted siRNA (siCryaa) (Genolution Pharmaceuticals, Seoul, South Korea) were introduced using Lipofectamine™ RNAiMAX (Invitrogen). EGFP reporter constructs were generated by cloning the 3′ untranslated region (3′UTR) fragments of *Cryaa* mRNA into the pEGFP-C1 vector (BD Bioscience, Heidelberg, Germany).

### Animals

Male albino Wistar rats, aged 4 weeks, were obtained from Orient Bio (Seongnam, South Korea) and housed in plastic cages in a climate-controlled facility maintained at 22 °C under a 12-h light/dark cycle with a relative humidity between 34 and 48%. Standard chow and water were provided ad libitum. All procedure of animal research was provided in accordance with the Laboratory Animals Welfare Act, the Guide for the Care and Use of Laboratory Animals and the Guidelines and Policies for Rodent experiment provided by the IACUC (Institutional Animal Care and Use Committee) in school of medicine, The Catholic University of Korea (Approval number: CUMC-2021–0144-03). This study was carried out in compliance with the ARRIVE guideline. IACUC and Department of Laboratory Animal (DOLA) in Catholic University of Korea, Songeui Campus accredited the Korea Excellence Animal Laboratory Facility from Korea Food and Drug Administration in 2017 and reaccredited​ in 2021. And also acquired AAALAC International full accreditation in 2018 and reaccredited​ in 2022.

To induce diabetes, a single intraperitoneal injection of streptozotocin (STZ; 60 mg/kg body weight; Sigma-Aldrich), dissolved in 0.05 M HCl-sodium citrate buffer (pH 5.5), was administered. Prior to the injection (designated as day one), the rats were rendered unconscious in a gas chamber with 2% isoflurane in oxygen. Following removal from the chamber, anesthesia was maintained using a mask that delivered 1.5% isoflurane in oxygen. Three days after the injection, blood samples were collected from the tail vein, and serum glucose levels were measured using an automated Accu-Check glucometer (Roche Diagnostics Corporation, Indianapolis, IN, USA). Rats exhibiting serum glucose levels greater than 250 mg/dL on day 3 were confirmed as diabetic and selected for further study. Body weight and serum glucose were monitored weekly following diabetes induction.

### Western blot analysis

Whole-cell extracts were obtained by lysing cells in RIPA buffer containing 10 mM Tris–HCl (pH 7.4), 150 mM NaCl, 1% NP-40, 1 mM EDTA, and 0.1% SDS. The resulting protein samples were separated using SDS-PAGE and transferred onto PVDF membranes (Millipore, Billerica, MA, USA). The membranes were then incubated with primary antibodies against HuD (1:1,000; Santa Cruz Biotechnology, Dallas, TX, USA; Cat# sc-28299), CRYAA (1:2,000; Santa Cruz Biotechnology; Cat# sc-28306), GFP (1:1,000; Santa Cruz Biotechnology; Cat# sc-9996), TNFα (1:200; Millipore; Cat# AB1837P), IL-1β (1:200; Abcam, Cambridge, MA, USA; Cat# ab9722), IL-6 (1:200; Abcam; Cat# ab9324), and β-actin (1:1,000; Abcam; Cat# ab8226). After primary antibody incubation, the membranes were treated with horseradish peroxidase (HRP)-conjugated secondary antibodies (Cell Signaling Technology, Beverly, MA, USA) and the immunoreactive bands were visualized using the WestGlow™ FEMTO ECL Chemiluminescent Substrate Kit (Biomax, Seoul, South Korea).

### RNA analysis and ribonucleoprotein immunoprecipitation (RNP-IP) analysis

Total RNA was extracted from whole cells using RNAiso™ Plus (TaKaRa, Shiga, Japan). The isolated RNA was reverse transcribed using the ReverTra Ace® qPCR RT Kit (Toyobo, Osaka, Japan), and transcript levels were subsequently quantified by real-time PCR using SensiFAST™ SYBR® No-ROX Mix (Bioline, MA, USA) with gene-specific primer sets (Table 1). RT-qPCR was carried out on a CFX Connect™ Real-Time System (Bio-Rad, CA, USA). For RNA immunoprecipitation (RIP) analysis, ribonucleoprotein complexes were immunoprecipitated with either anti-HuD antibody or control IgG (Santa Cruz Biotechnology) [11]. Briefly, after immunoprecipitation, the complexes were treated with DNase I and proteinase K, and the RNA isolated from these samples was analyzed by RT-qPCR using the primers listed in Table 1.

**Table 1:** Primer sequences used in this study

Primers for EGFP-reporter	Sequences		
rat *Cryaa*-3U-F	5'- AAAAAGATCTTAAGCAGGCCTCGCCTTGG −3'		
rat *Cryaa*-3U-R	5'- AAAAGGTACCGCTTGTCACCTGCTCT −3'		
**Primers for PCR**	**Sequences**	**Product size**	**References (primer bank number)**
rat *HuD*-F	5'- GCCTCAGGTGTCAAATGGACC −3'	243 bp	*N.S*
rat *HuD*-R	5'- CCATACCCTAAACTCTGTCCTGT −3'		
rat *Cryaa*-F	5'- CCTGCTGCCCTTCCTGTCGT −3'	210 bp	*N.S*
rat *Cryaa*-R	5'- TCCTGGCGCTCGTTGTGCT −3'		
rat* IL-1β*-F	5'- CTCACAGCAGCATCTCGACAAGAG −3'	95 bp	doi.org/10.1155/2021/6657673
rat *IL-1β*-R	5'- TCCACGGGCAAGACATAGGTAGC −3'		
rat *IL-6*-F	5'- TCCTACCCCAACTTCCAATGCTC −3'	79 bp	J Hazard Master (2015) 287;392
rat *IL-6*-R	5'- TTGGATGGTCTTGGTCCTTAGCC −3'		
rat *Tnfα*-F	5'- CCAGGTTCTCTTCAAGGGACAA −3'	80 bp	doi.org/10.1155/2021/6657673
rat *Tnfα*-R	5'- GGTATGAAATGGCAAATCGGCT −3'		
rat *Gapdh*-F	5'- TGCCACTCAGAAGACTGTGG −3'	123 bp	J Neurosci (2010) 30;15,007
rat *Gapdh*-R	5'- TTCAGCTCTGGGATGACCTT −3'		

### Immunofluorescence

Cryosections of the eyes were allowed to air-dry and then rinsed twice with phosphate-buffer saline (PBS). The sections were incubated in 0.5% hydrogen peroxide prepared in methanol for 30 min, followed by two additional PBS washes. To prevent nonspecific antibody binding, the sections were blocked with 2% bovine serum albumin (BSA) in PBS for 1 h at room temperature. Slides were then incubated overnight at 4 °C with primary antibodies against HuD and CRYAA, diluted in 2% BSA. After three washes with PBS (5 min each), the sections were incubated with fluorophore-conjugated secondary antibodies (diluted in PBS) for 1 h at room temperature in the dark. Nuclei were counterstained with 4′,6-diamidino-2-phenylindole (DAPI), and the slides were mounted using fluorescence mounting medium. Images were acquired using a confocal laser scanning microscope (LSM 900; Carl Zeiss, Oberkochen, Germany).

### Cell viability and apoptosis assays

Cell viability was determined by evaluating the reduction of MTT (3-(4,5-dimethylthiazol-2-yl)−2,5-diphenyltetrazolium bromide; Invitrogen). R-28 cells were plated in 96-well plates and cultured for 24 h in a humidified incubator with 5% CO₂ at 37 °C. After incubation, MTT solution (5 mg/mL) was added to each well, and the cells were further incubated at 37 °C for 2 h. The medium was then removed, and formazan crystals were dissolved by adding 100 µL of 0.04 N acidified isopropanol along with 100 µL of distilled water. The plates were agitated for 10 min, and absorbance was measured at 570 nm using a microplate reader (SpectraMax 190; Molecular Devices, San Jose, CA, USA).

To assess apoptosis, caspase-3/7 activity was measured using the Caspase-Glo® 3/7 Assay kit (Promega, Madison, WI, USA) according to the manufacturer’s protocol. After treatment, cells were incubated with the Caspase-Glo® reagent in a 1:1 ratio, and luminescence was measured after 1 h at room temperature in the dark using a luminometer (Spectra Max L; Molecular Devices).

### Statistical analysis

Each experiment was independently repeated at least three times, and all the samples were tested in triplicate. Data are expressed as the mean ± standard error of the mean (SEM). Statistical comparisons were made using an unpaired Student’s t-test, and a *p-value* below 0.05 was considered statistically significance.

## Results

### HuD expression in neuro-retinal cells

**Fig. 1:**
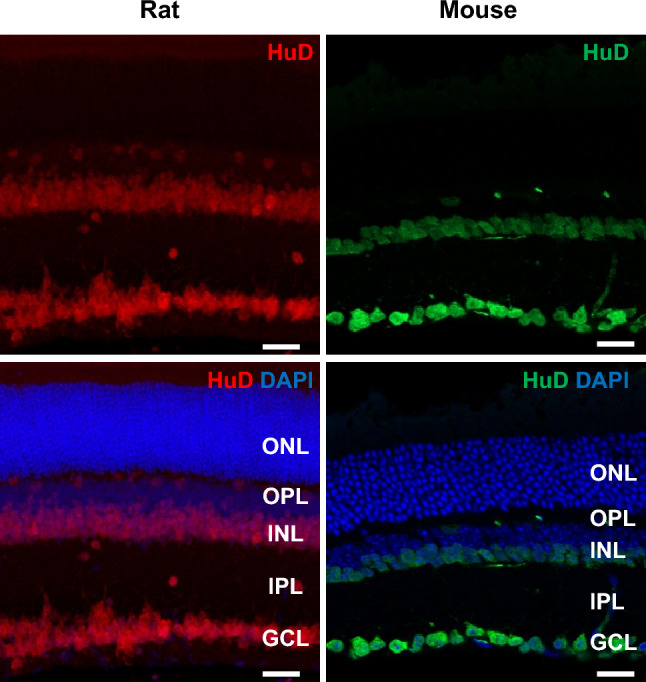
HuD is expressed in neuro-retinal cells. The expression of HuD was analyzed by immunostaining in the retinas of rats and mice. Nuclei were counterstained with DAPI. Ganglion cell layer (GCL), inner nuclear layer (INL), inner plexiform layer (IPL), outer plexiform layer (OPL), and outer nuclear layer (ONL). Scale bar, 20 µm

The roles of HuD have been revealed in neuronal function, maintenance, and development, owing to its predominant expression in neuronal cells [9, 10]. Since the role of HuD in insulin synthesis has been reported [16], its function has also been studied in neuro-endocrinal β cells such as autophagy, lipid metabolism, cell cycle, and mitochondrial dynamics [17]. However, the role of HuD in the retina has not been examined. To determine whether HuD functions in the retina, we first examined the presence of HuD expression in retinal tissues by immunostaining. As shown in Fig. 1, HuD was predominantly expressed in ganglion cells and the inner nuclear layers of both rat and mouse retinal tissues. These findings suggest that, unlike its role in neural and neuroendocrine cells, HuD may have a distinct and important function in neuro-retinal cells.

### Down-regulations of HuD and CRYAA in the retinas of STZ-induced diabetic rats

**Fig. 2:**
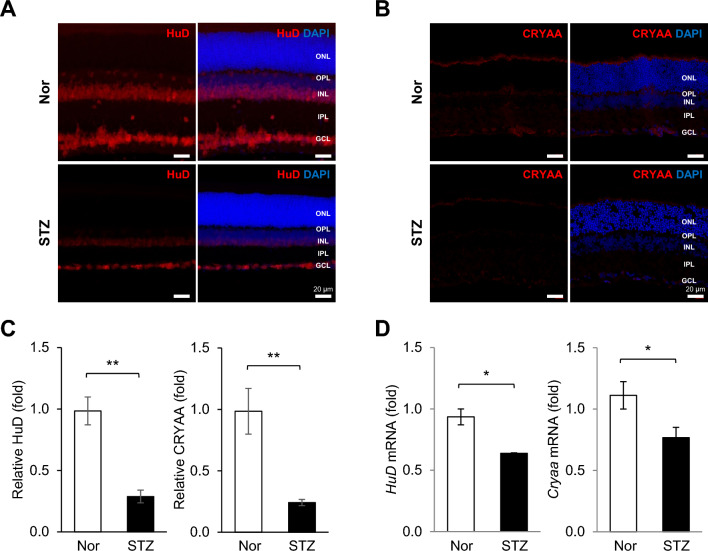
HuD and CRYAA are down-regulated in neuro-retinal cells of diabetic rats. **A** and **B** The expression levels of HuD and CRYAA were analyzed by immunofluorescence microscope in the retinas of normal (Nor) and Streptozotocin-induced diabetic (STZ) rats at 4 weeks post-injection (n = 6 for Nor; n = 6 for STZ). Ganglion cell layer (GCL), inner nuclear layer (INL), inner plexiform layer (IPL), outer plexiform layer (OPL), and outer nuclear layer (ONL). Scale bar, 20 µm. **C** Quantification of immunofluorescence intensity in Nor and STZ groups. **D** The relative levels of *HuD* and *Cryaa* mRNAs in retinal tissues from Nor- and STZ-rats were analyzed by RT-qPCR. *Gapdh* mRNA was used for normalization. Data represent the means ± SEM from three independent experiments. *, *p* < *0.05*; **, *p* < *0.01*

It is well-established that HuD expression is down-regulated in β cells of diabetic mice (*db/db*) [18]. These findings led us to hypothesize that HuD may also be regulated in retinal cells under diabetic conditions. To investigate this possibility, we examined HuD expression in the retinas of normal and STZ-induced diabetic rats (STZ-rat). As shown in Fig. 2A, HuD expression was significantly down-regulated in both the GCL and INL of the retina in STZ-rats compared to normal rats. Additionally, the *HuD* mRNA level was also decreased in the retinal tissues of STZ-rats (Fig. 2D). Interestingly, we observed parallel decreases in the protein and mRNA levels of CRYAA in the retinas of STZ-rats (Fig. 2B and D). These findings collectively implicate the down-regulation of HuD and CRYAA in the pathophysiology of diabetic retinal degeneration.

### Down-regulations of HuD and CRYAA in neuro-retinal cells by high glucose

**Fig. 3:**
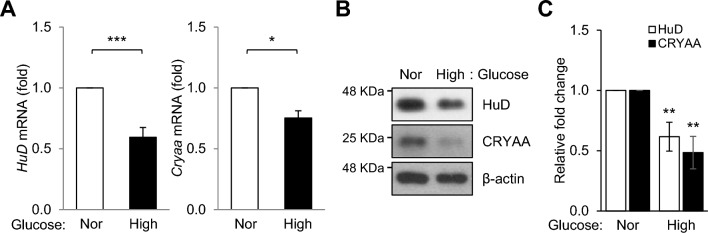
HuD and CRYAA are down-regulated in neuro-retinal cells by high glucose. After incubation of R-28 cells with 5.5 or 25 mM of glucose for 72 h, the mRNA and protein levels of HuD and CRYAA were analyzed by RT-qPCR (**A**) and Western blotting (**B**), respectively. **C** Quantification of protein expression levels. *Gapdh* mRNA was used for normalization, and β-actin was used as a loading control. Data represent the means ± SEM from three independent experiments. Images (**B**) are representative from three independent experiments. *p < *0.05*, **p < *0.01*, ***p < *0.001*

The diminished levels of HuD and CRYAA in the retinas of diabetic rats strongly suggest that their expression may be influenced by glucose levels. To investigate this possibility, we exposed neuro-retinal R-28 cells to high glucose (25 mM) for 72 h and performed RT-qPCR and Western blotting analyses. As expected, both *HuD* and *Cryaa* mRNA and protein expression levels were significantly down-regulated in neuro-retinal cells exposed to high glucose (Fig. 3A and B). Therefore, these results underscore the potential of HuD and CRYAA to regulate the crucial functions of neuro-retinal cells under hyperglycemic conditions.

### Regulation of *Cryaa* mRNA expression by HuD via its 3′-untranslated region binding

**Fig. 4:**
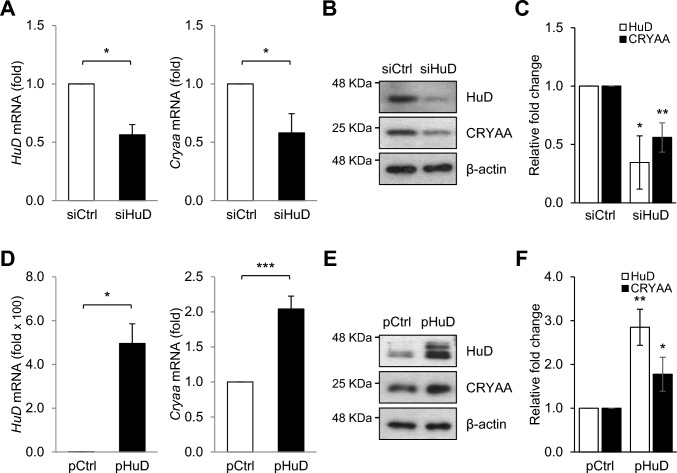
HuD positively regulates CRYAA expression in neuro-retinal cells. Forty-eight hours after transfection of R-28 cells with either of HuD siRNA (siHuD) or the HuD overexpression plasmid (pHuD) along with the appropriate controls (siCtrl and pCtrl, respectively), levels of *HuD* and *Cryaa* mRNA and protein were assessed by RT-qPCR **A** and **D** and Western blotting **B** and **E**, respectively. **C** and **F** Quantification of protein expression levels. *Gapdh* mRNA was used for normalization, and β-actin was used as a loading control. Data represent the means ± SEM from three independent experiments. Images in (**B** and **E**) are representative from three independent experiments. *p < *0.05*, **p < *0.01*, *** *p* < *0.001*

**Fig. 5:**
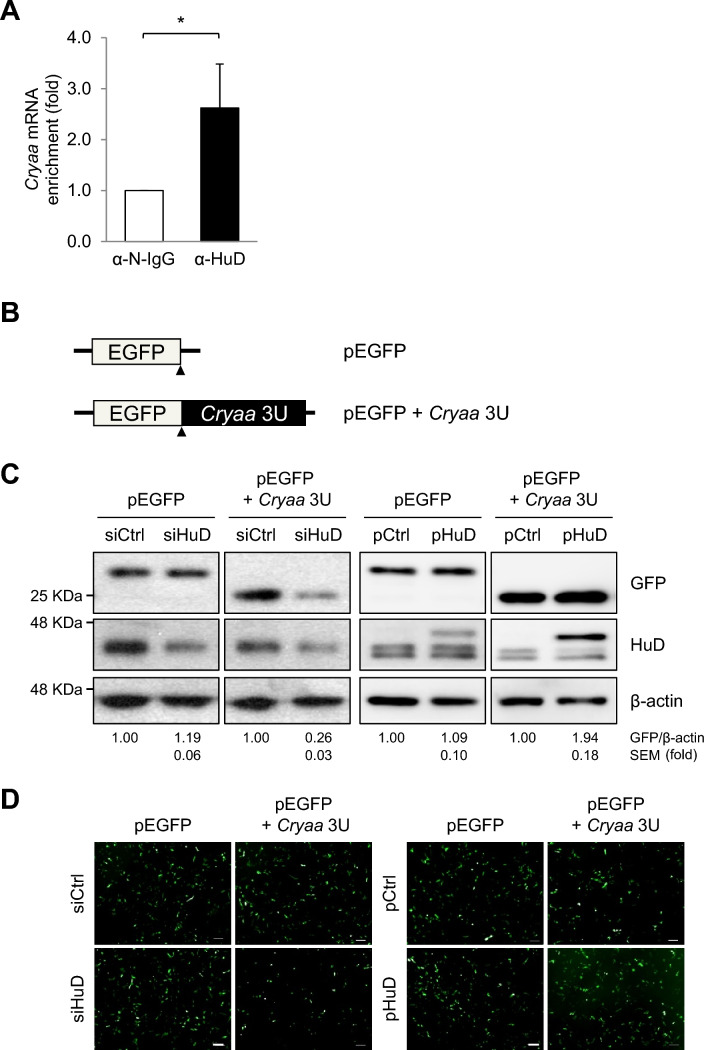
HuD regulates the expression of *Cryaa* mRNA by binding to its 3’UTR. **A** The RNP complex in R-28 cells was isolated by immunoprecipitation using anti-HuD antibody and enriched *Cryaa* mRNA in HuD-IP was analyzed by RT-qPCR. Data represent the means ± SEM from three independent experiments. *p < *0.05*. **B** Schematic of reporter plasmids, 3’UTR region of *Cryaa* mRNA (3U) was inserted into the pEGFP vector. **C** After transfection of HuD siRNA or the overexpression plasmid along with appropriate controls, R-28 cells were sequentially transfected with reporter plasmids. Relative expressions of GFP and HuD were analyzed by western blotting. Relative density for the indicated protein is shown on the *bottom*. β-actin was used as a loading control. **D** Immunofluorescence images of GFP in transfected cells. Data represent means ± SEM from three independent experiments

Next, we tested whether HuD is an upstream regulator of CRYAA expression in neuro-retinal cells by transient silencing or overexpressing *HuD* and then measuring *Cryaa* mRNA and protein levels using RT-qPCR and Western blotting analysis, respectively. *HuD* silencing reduced both *Cryaa* mRNA and protein levels (Fig. 4A and B), whereas *HuD* overexpression induced this effect (Fig. 4D and E), suggesting that HuD regulates the stability and translation of *Cryaa* mRNA. To further elucidate the regulation of *Cryaa* mRNA by HuD, we performed RNP-IP using an anti-HuD antibody. As shown in Fig. 5A, the enriched *Cryaa* mRNA was assessed in the HuD-bound RNP complex. These results indicate that *Cryaa* mRNA has a binding site for HuD on its nucleic acid sequence. HuD is a turnover- and translation-regulatory RNA-binding protein that regulates the expression of target mRNAs by binding to their 3′UTR. To further investigate whether HuD directly influences *Cryaa* mRNA, we constructed EGFP reporter vectors by cloning the 3’UTR of *Cryaa* mRNA (Fig. 5B). EGFP reporter plasmids were transfected into neuro-retinal cells, with *HuD* either silenced or overexpressed. Relative GFP expression was then analyzed via Western blotting (Fig. 5C). *HuD* silencing specifically down-regulated GFP expression in cells transfected with pEGFP + *Cryaa* 3U, but not in those with control reporter (pEGFP). Conversely, *HuD* overexpression up-regulated GFP expression in pEGFP + *Cryaa* 3U (Fig. 5C and D). These results collectively suggest that HuD binds to the 3′UTR of *Cryaa* mRNA to regulate its expression.

### Inflammatory cytokine expression by HuD-CRYAA axis in neuro-retinal cells

**Fig. 6:**
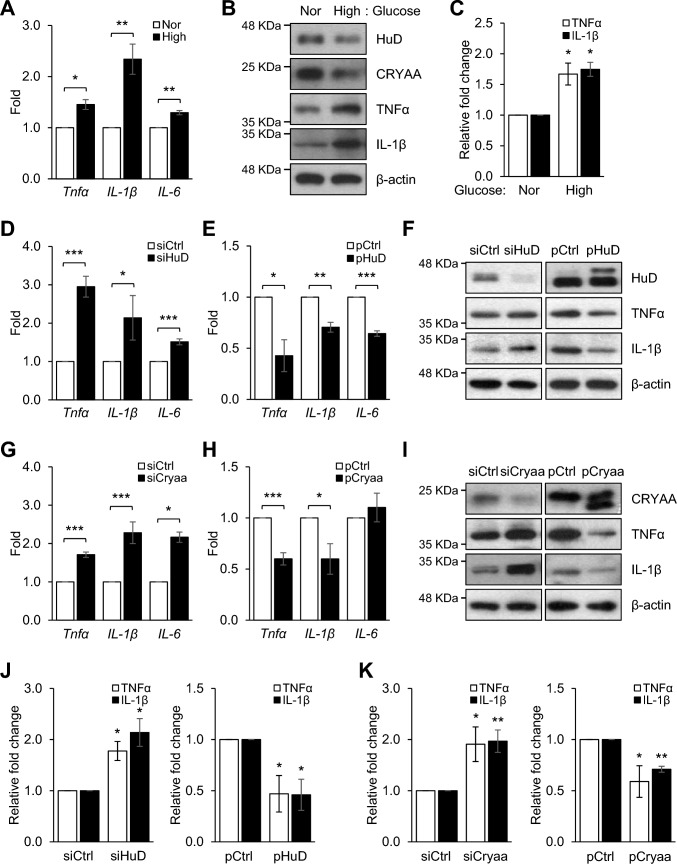
Neuro-retinal inflammation is regulated by HuD-CRYAA axis on high glucose. **A** and **B** After incubation of R-28 cells with 5.5 and 25 mM of glucose for 72 h, mRNA levels of *Tnfα, IL-1β*, and *IL-6* were analyzed by RT-qPCR (**A**), as well as protein levels of HuD, CRYAA, TNFα, and IL-1β by western blotting (**B**). **D**–**I** After transfection of R-28 cells with either of *HuD* or *Cryaa* siRNAs (siHuD or siCryaa) or *HuD* or *Cryaa* overexpression plasmids (pHuD or pCryaa) along with appropriate controls (siCtrl and pCtrl, respectively), the mRNA levels of *Tnfα*, *IL-1β*, and *IL-6* were analyzed by RT-qPCR (D, E, G, and H), as well as protein levels of HuD, CRYAA, TNFα, and IL-1β by western blotting (**F** and **I**). **C**, **J**, and **K** Quantification of western blot data. *Gapdh* mRNA was used for normalization, and β-actin was used as a loading control. The images in (B, F, and I) are representative from three independent experiments. Data represent the means ± SEM from three independent experiments. *p < *0.05*, **p < *0.01*, ***p < *0.001*

Abundant evidence indicates that inflammation contributes to the development of DR [19]. The levels of several inflammatory cytokines, such as TNF-α, IL-1β, and IL-6, are elevated in the vitreous, aqueous humor, and ocular tissues of diabetic patients with DR [20]. We investigated whether diabetic conditions induce the expression of inflammatory cytokines in neuro-retinal cells. As shown in Fig. 6A, the expressions of *Tnf-α, IL-1β,* and *IL-*6 mRNAs were upregulated in R-28 cells under high glucose treatment. Moreover, the protein expressions levels of TNF-α and IL-1β increased, coinciding with the decreased levels of HuD and CRYAA (Fig. 6B). We further investigated the involvement of HuD in the regulation of inflammatory cytokines in neuro-retinal cells. R-28 cells were transfected with siRNA targeting *HuD* (siHuD) or overexpression plasmids for *HuD* (pHuD) for 48 h, after which the relative levels of *Tnf-α*, *IL-1β*, and *IL-6* mRNAs were assessed by RT-qPCR. As shown in Fig. 6D and E, *HuD* silencing induced the expression of all inflammatory cytokines in neuro-retinal cells, whereas their levels were reduced by *HuD* overexpression. Additionally, we examined whether CRYAA plays a role in the elevated expression of inflammatory cytokines in R-28 cells (Fig. 6G and H). As expected, *Cryaa* silencing significantly increased the levels of *Tnf-α*, *IL-1β*, and *IL-6* mRNAs in neuro-retinal cells, whereas *Cryaa* overexpression reduced their expressions. Furthermore, the levels of TNF-α and IL-1β proteins were concordant with mRNA expressions upon *HuD* and *Cryaa* silencing and overexpression (Fig. 6F and I). Collectively, these results suggest that the reduction in HuD and CRYAA levels under diabetic conditions contributes to elevated inflammatory cytokine levels during the development of DR.

### Alleviative role of HuD and CRYAA in neuro-retinal cell viability.

**Fig. 7:**
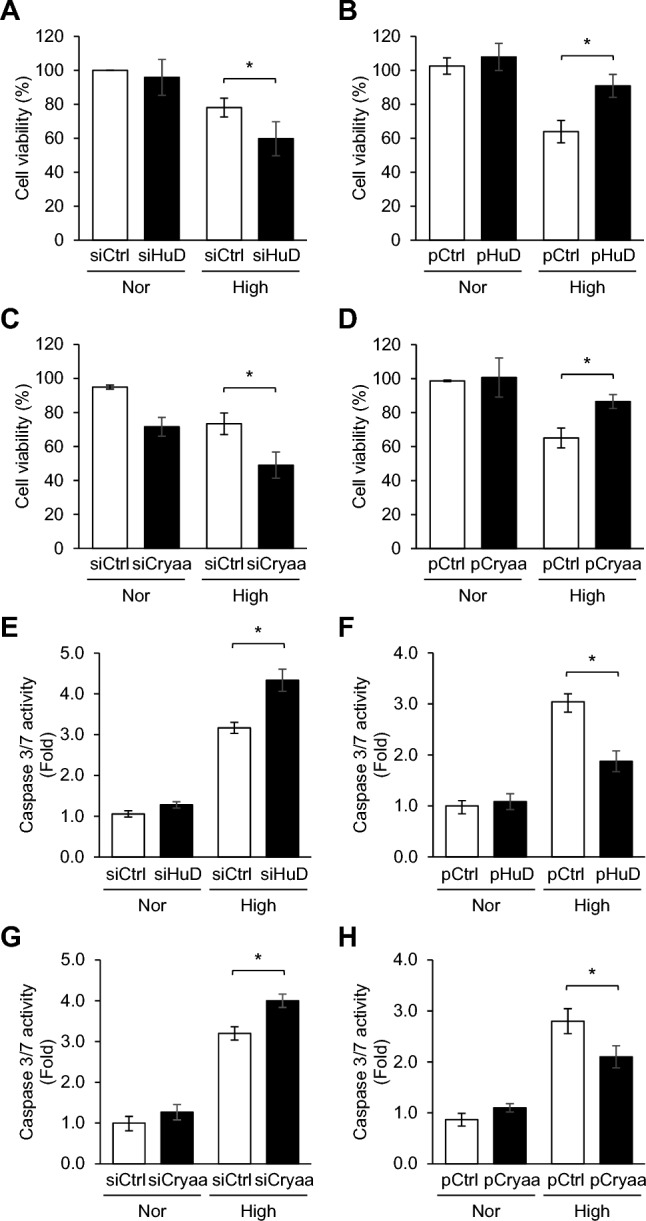
HuD and CRYAA alleviate high glucose-induced R-28 cell death. After transfection with *HuD* or *Cryaa* siRNA (siHuD or siCryaa) (**A**, **C**, **E**, and **G**) or *HuD* or *Cryaa* overexpression plasmids (pHuD or pCryaa) (**B**, **D**, **F**, and **H**) along with appropriate controls (siCtrl or pCtrl), R-28 cells were incubated with 5.5 or 25 mM of glucose for 72 h, followed by cell viability assay using MTT assay (**A**–**D**) and caspase-3/7 activity apoptosis assay (**E**–**H**). Data represent the means ± SEM from three independent experiment. *p < *0.05*

Hyperglycemia induces oxidative stress, which affects various aspects of cell viability. To assess the influence of HuD and CRYAA on neuro-retinal cell viability, R-28 cells were transfected with siHuD or siCryaa and then exposed to high glucose for 72 h, followed by MTT analysis. Although *HuD* and *Cryaa* silencing had no effect on cells under normal glucose conditions, the reduction in R-28 cell viability was exacerbated by *HuD* and *Cryaa* silencing (Fig. 7A and C) under high glucose condition. To elucidate the role of HuD and CRYAA in mitigating the decrease in cell viability caused by high glucose levels, cell viability assays were conducted by transfecting R-28 cells with overexpression plasmids for *HuD* (pHuD) or *Cryaa* (pCRYAA). Intriguingly, overexpression of *HuD* and *Cryaa* alleviated the decrease in neuro-retinal cell viability induced by high glucose (Fig. 7B and D). In addition, we evaluated apoptosis levels using caspase-3/7 activity assays. As shown in Fig. 7E and G, knockdown of *HuD* and *Cryaa* under high glucose conditions significantly increased caspase-3/7 activity in R-28 cells. In contrast, overexpression of *HuD* and *Cryaa* markedly reduced caspase-3/7 activity under the same conditions (Fig. 7F and H), demonstrating their protective roles against hyperglycemia-induced apoptotic cell death. Taken together, our findings indicate that modulating the HuD-CRYAA pathway is essential for both the survival and proper function of neuro-retinal cells as diabetic retinopathy develops.

## Discussion

Although microvascular complications are a major consideration in DR, neuro-retinal degeneration is recognized as the leading cause of vision loss. Several studies have reported that neuro-retinal damage with structural changes is priorly observed in the retina of patients with diabetes without any signs of DR. Barber et al*.* demonstrated a decrease in the GC-IPL [21], and Carpineto et al*.* showed a significant reduction in the GC-IPL and retinal nerve fiber layer thickness in type 2 diabetes patients with no DR or mild NPDR [22]. Although these reports suggest that ganglion cells are the primary cells influenced by neuro-retinal degeneration developed at the early stage of DM before DR diagnosis, the precise molecular mechanism leading to the decrease in ganglion cells has not yet been fully understood. In this study, we demonstrated that an RNA binding protein, HuD is expressed in neuro-retinal cells, specifically in ganglion cells, and plays a novel role in neuro-retinal degeneration via post-transcriptional regulation of *Cryaa* mRNA. Additionally, we observed a significant decrease in both HuD and CRYAA levels in the GCL of STZ-induced diabetic rats. Deficiencies in these proteins were implicated in the reduced viability of neuro-retinal cells under hyperglycemic conditions. These results suggest that both HuD and CRYAA have potential roles in the pathological processes of neuro-retinal degeneration during the early stage of diabetes.

**Fig. 8:**
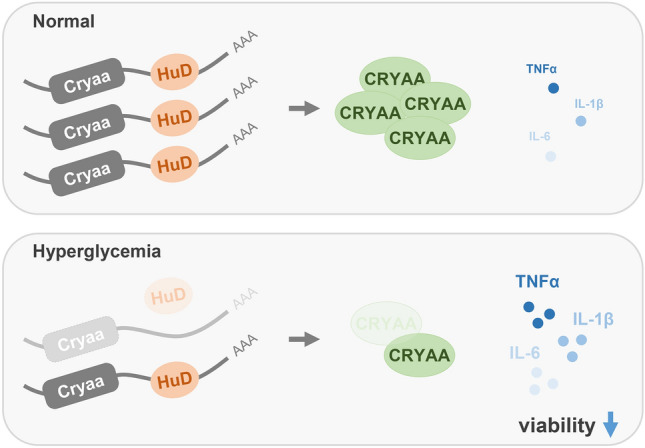
Schematic diagram of HuD-mediated regulation of CRYAA in neuro-retinal cells under hyperglycemic conditions

Crystallins were first identified as major structural components of the lens [23]. Additionally, alpha-crystallins are also present in various parts of the eye, including the retina, cornea, optic nerve, as well as in glial cells like astrocytes and Müller cells. [24]. Alpha-crystallins possess a chaperone-like function, preventing apoptosis by avoiding aberrant protein interaction and degradation [25]. Several studies have reported a reduction in alpha-crystallin levels in various retinal degenerations, leading to reduced survival of retinal ganglion cells [26]. Although the up-regulation of CRYAA has been observed in the eyes of humans with diabetes [27], STZ-induced diabetic rats [28], high fat/STZ-induced diabetic rats [29], and Ins2^*Akita*^ diabetic mice [30], several studies have also reported diminished levels of CRYAA in diabetic retinas. Kim et al*.* determined that the levels of *Cryaa* mRNA and protein were significantly reduced in the retina of STZ-induced diabetic mice [13]. Moreover, down-regulated transcriptional level and translational efficiency of *Cryaa* mRNA have also been demonstrated in the retinas of STZ-induced diabetic rat models [14, 15]. In the present study, we observed decreased levels of CRYAA in the retinal GCL of STZ-induced diabetic rats (Fig. 2B). Furthermore, CRYAA knock-down promoted a reduction in neuro-retinal cell viability under high glucose treatment (Fig. 7C). These findings strongly suggest that decreased levels of CRYAA are closely associated with the viability of neuro-retinal cells under hyperglycemic conditions, indicating the potential of CRYAA as a specific indicator for diagnosing neuro-retinal degeneration in the early stage of diabetes (Fig. 8).

Alpha-crystallins prevent apoptosis induced by several factors, such as etoposide, staurosporine, and sorbitol [31], UV [32], TNFα [33], hydrogen peroxide [34], and okadaic acid [35]. They inhibit apoptosis by directly interacting with caspase-3, Bcl-x(s) and Bax [31, 36], upregulating PI3 kinase activity [25], as well as regulating PKCα, RAF/MEK, ERK, and AKT signaling [32]. The reduced chaperone function of alpha-crystallin due to mutation has been shown to result in a loss of its protective ability against apoptosis [37]. Moreover, the absence of alpha-crystallin has been demonstrated to enhance retinal degeneration in chemically induced hypoxia [38] and in sodium iodate-induced AMD models [39]. Conversely, alpha-crystallin has been shown to improve the viability of retinal ganglion cells [40] and optic nerve axons after optic nerve crush [41]. Additionally, intravitreal injection of CRYAA via an adenovirus ameliorated apoptotic cell death and vascular leakage in the diabetic retina [13]. In this study, we further demonstrated that overexpression of CRYAA alleviated the viability of neuro-retinal cells under high glucose conditions (Fig. 7D), indicating that restoring CRYAA expression under hyperglycemic conditions might be an effective therapeutic strategy for preventing neuro-retinal cell degeneration at the early stage of diabetes.

The protective function of CRYAA is regulated by various regulatory factors. The expression of crystallins is controlled by several transcription factors including Pax-6, Maf, Sox, neural retina leucine zipper (NLR), retinoic acid receptors (RARs), Prox1, Six3, γFBP-B, HSF2, HSF4, CREB, and AP-1 [42–46]. Additionally, alpha-crystallins are regulated by phosphorylation via cyclic adenosine monophosphate (cAMP) and dephosphorylation [47, 48]. It has also been reported that the chaperone activity of alpha-crystallin is regulated by glycation and acetylation [49–51]. That is, understanding the detailed regulatory mechanism for CRYAA expression and its chaperone activity is necessary to establish therapeutic strategies targeting the degeneration of neuro-retinal cells. In this study, we verified that HuD is a crucial upstream regulator of *Cryaa* mRNA. Using RNP-IP and EGFP-reporter analyses, we demonstrated that the expression CRYAA is controlled by binding between HuD and the 3′UTR of *Cryaa* mRNA (Fig. 5). HuD silencing decreased the levels of *Cryaa* mRNA and protein, whereas HuD overexpression significantly increased these levels in neuro-retinal R-28 cells. Although our results suggest that HuD is a positive post-transcriptional regulator for *Cryaa* mRNA, further clarification is needed to elucidate the precise post-transcriptional mechanisms underlying its regulatory role.

HuD is an RNA binding protein predominantly expressed in neuronal cells. Increasing evidence suggests that HuD plays crucial roles in several types of endocrine and cancer cells [17]. Although some studies have reported the direct or indirect regulation of HuD during neuronal and endocrine disease development, the molecular mechanisms controlling *HuD* gene expression have not yet been fully examined. Three molecular pathways regulating the function and abundance of HuD have been elucidated: protein kinase C (PKC) [52, 53], coactivator associated arginine methytransferase 1 (CARM1) [54], and protein kinase B (PKB/AKT) [55]. Other studies have determined the transcriptional and post-transcriptional control of *HuD* gene expression. The expression of *HuD* mRNA is upregulated during neuronal differentiation by thyroid hormone (T3) [56], and Ngn2 has been identified as a transcription factor responsible for increasing *HuD* mRNA expression [57]. Forkhead box O1 (Foxo1) negatively affects the transcription of *HuD* in pancreatic β cells under low glucose conditions [16], and miR-375 also negatively controls both the stability and translation of *HuD* mRNA [58]. Special adenine–thymine (AT)-rich DNA-binding protein 1 (SATB1) was recently identified as an activator of the *HuD* promoter during neuronal differentiation [59]. Here in, we revealed the presence of HuD expression in neuro-retinal ganglion cells (Fig. 1). Furthermore, HuD expression was found to be regulated under hyperglycemic conditions, including in the retinas of STZ-induced diabetic rats and neuro-retinal cells under high glucose treatment. While these findings suggest that HuD may be an essential factor for maintaining the homeostasis of neuro-retinal cells, further elucidation of the molecular regulatory mechanism of HuD expression is needed to protect neuro-retinal cells from hyperglycemia-induced degeneration.

Inflammation is a significant feature in DR pathogenesis observed in both animal models and patients with diabetes. Elevated glucose levels lead to metabolic dysfunction, oxidative stress, and the production of reactive oxygen species, resulting in inflammatory responses. Several studies have demonstrated increased levels of pro-inflammatory cytokines, including TNFα, IL-1β, and IL-6, in the serum, vitreous and aqueous humor, as well as in the retinal tissues of patients with DR [60]. Up-regulated pro-inflammatory cytokines promote inflammatory responses through various pathways, leading to neuro-retinal degeneration such as cell apoptosis. In this study, we demonstrated increased levels of TNFα, IL-1β, and IL-6 in neuro-retinal cells under high glucose treatment and found that their levels were negatively regulated by both HuD and CRYAA expression. These results suggest that HuD and CRYAA have the potential to inhibit inflammatory responses, thereby alleviating cell death. However, further studies are needed to determine whether *Tnfα*, *IL-1β*, and *IL-6* mRNAs are molecular targets of HuD and how their expression is regulated by CRYAA to prevent neuro-retinal inflammatory responses in DR.

Our study has several limitations. First, the use of the R-28 cell line may not fully recapitulate the complexity of primary neuro-retinal cells or the in vivo retinal environment. Second, our diabetic model relies on STZ-induced diabetes in rats. Although STZ-induced diabetic rat models are widely used, they may not perfectly mimic human diabetic retinopathy. Therefore, alternative diabetic animal models, such as mouse models (e.g., Ins2^Akita^, NOD, Lepr^db^, Kimba, or Akimba) or rat models (e.g., ZDF, OLETF, BB, WBN/Kob, SDT, or GK), should be considered to further support the protective role of the HuD/CRYAA axis against high glucose-induced neuro-retinal degeneration. Third while our data indicate that HuD binds to the 3′UTR of *Cryaa* mRNA, it does not fully distinguish whether HuD primarily affects mRNA stability, translation efficiency, or both. Further mechanistic studies, such as mRNA decay assays or ribosome profiling, are necessary to clarify this point. Finally, although the overexpression experiments suggest that enhancing HuD/CRYAA levels can be protective, the study does not evaluate this protective role in vivo. Additional in vivo experiments are needed to further assess the therapeutic potential of targeting this pathway.

In summary, we propose a novel molecular mechanism and role of the HuD/CRYAA axis in neuro-retinal degeneration during the early stages of diabetes. Our results indicated that HuD expression is down-regulated in neuro-retinal cells under hyperglycemic conditions, leading to a decrease in CRYAA expression through post-transcriptional regulation. Downregulation of HuD and CRYAA is highly implicated in cellular inflammation, affecting neuro-retinal cell viability under hyperglycemic conditions. Restoration of HuD and CRYAA expression is beneficial for improving neuro-retinal cell survival and overcoming neuro-retinal degeneration in DR.

## Data Availability

The data that support the findings of this study are available from the corresponding author upon reasonable request.

## Abbreviations

AREs: Adenylate/uridylate-rich elements
CRYAA: Alpha-crystallin A
DR: Diabetic retinopathy
EGFP: Enhanced green fluorescence protein
GCL: Ganglion cell layer
GC-IPL: Ganglion cell-inner plexiform layer
HuD: Hu-antigen D
INL: Inner nuclear layer
NPDR: Non-proliferative diabetic retinopathy
PDR: Proliferative diabetic retinopathy
RGC: Retinal ganglion cell
RNP: Ribonucleoprotein
STZ: Streptozotocin
